# Prediction model of gastrointestinal tumor malignancy based on coagulation indicators such as TEG and neural networks

**DOI:** 10.3389/fimmu.2025.1507773

**Published:** 2025-03-25

**Authors:** Fulong Yu, Chudi Sun, Liang Li, Xiaoyu Yu, Shumin Shen, Hao Qiang, Song Wang, Xianghua Li, Lin Zhang, Zhining Liu

**Affiliations:** ^1^ Department of General Surgery, The Second Affiliated Hospital of Anhui Medical University, Hefei, Anhui, China; ^2^ School of Computer Science and Technology, Soochow University, Suzhou, Jiangsu, China; ^3^ Clinical Pharmacy, School of Pharmacy, Wannan Medical College, Wuhu, Anhui, China; ^4^ Department of Oncology, The Second Affiliated Hospital of Anhui Medical University, Hefei, Anhui, China; ^5^ Department of Molecular Pathology, Hefei Da’an Medical Laboratory Co., Ltd., Hefei, Anhui, China; ^6^ The School of Public Health and Preventive Medicine, Monash University, Melbourne, VIC, Australia

**Keywords:** TEG, machine learning, predictive models, gastric cancer, colorectal cancer, coagulation indicators

## Abstract

**Objectives:**

Accurate determination of gastrointestinal tumor malignancy is a crucial focus of clinical research. Constructing coagulation index models using big data is feasible to achieve this goal. This study builds various prediction models through machine learning methods based on the different coagulation statuses under varying malignancy levels of gastrointestinal tumors. The aim is to use coagulation indicators to predict the malignancy of gastrointestinal tumors, expand the methods and ideas for coagulation index tumor prediction, and identify independent risk factors for gastrointestinal tumor malignancy.

**Methods:**

Clinical data of 300 patients with gastrointestinal diseases were collected from the Second Affiliated Hospital of Anhui Medical University from January 2024 to August 2024 and grouped according to TNM and G staging, representing tumor malignancy levels. First, independent influencing factors of gastrointestinal tumor malignancy were identified using stepwise multivariate logistic regression. ROC curves were used to assess the ability of TEG five items and other coagulation indicators to distinguish between malignancy levels of gastrointestinal tumors. Finally, we constructed a network model suitable for our task data based on residual networks, named the Residual Fully Connected Binary Classifier (RFCBC). This model was compared with other commonly used binary classification methods to select the optimal model.

**Results:**

The TEG five items (AUC values: R: 0.682; K: 0.731; α-angle: 0.736; MA: 0.699; CI: 0.747) showed better discrimination ability in the G group than other coagulation indicators. Although the TNM group showed moderate discrimination ability, it did not exhibit a significant advantage over other indicators. The R and MA values were identified as independent influencing factors in both TNM and G groups. Ultimately, the RFCBC prediction model showed the best predictive performance compared to other binary classification machine learning models (TEG five items: 87.56%; Thromboelastogram et al.: 88.6%).

**Conclusion:**

This study found that the R and MA values are independent predictive factors for the malignancy of gastrointestinal tumors. Compared to other coagulation indicators, the TEG five items have better discrimination ability regarding tumor malignancy. The RFCBC model created in this study outperforms other commonly used binary classification methods in predicting the malignancy of gastrointestinal tumors, providing a new model construction method and feasible approach for future coagulation index prediction of gastrointestinal tumor malignancy.

## 1 Background

Gastrointestinal malignant tumors are among the most common malignant tumors worldwide and rank as the second leading cause of cancer-related deaths globally, following lung cancer (1). In China, the incidence and mortality rates of gastrointestinal malignancies are gradually increasing (2). Early symptoms are often not prominent, and by the time of discovery, the disease is usually at the middle to late stage, often accompanied by symptoms such as intestinal obstruction, changes in bowel habits, changes in stool characteristics, and weight loss anemia, leading to delayed treatment (3). Therefore, early detection and prediction of tumor malignancy have become a key focus of research (4).

Hypercoagulability has long been considered a characteristic of gastrointestinal malignancies (5, 6). Patients with malignant tumors exhibiting hypercoagulability have shorter overall and disease-free survival postoperatively compared to patients with normal coagulation (7). Traditional coagulation function tests, such as prothrombin time (PT), activated partial thromboplastin time (APTT), and fibrinogen (Fig) (8), have been used for relevant examinations in malignant tumors. However, traditional coagulation function tests have limitations as they only reflect the static characteristics of plasma coagulation at a specific time point (9). Thromboelastography (TEG) indicates the dynamic changes in blood coagulation. It includes seven parameters: R-value, K-value, α-angle, MA-value, CI, LY30, and EPL. In 1948, Hertert first described TEG as a method for real-time evaluation of the viscoelastic properties of whole blood. In recent years, TEG has been extensively used for detecting hypercoagulability related to cancer, such as lung cancer (10), liver cancer (11), and breast cancer (12).

Due to differences in tumor cell malignancy and staging, gastrointestinal malignant tumor patients exhibit varying coagulation statuses under different malignancy levels and stages. TEG can comprehensively assess the activity of coagulation factors, fibrinogen function, platelet function, and fibrinolysis status. Therefore, we grouped the study population into benign and malignant gastrointestinal tumor groups, further subdividing the malignant tumor group based on postoperative pathology into TNM and G stages. We collected TEG and other coagulation indicators for a retrospective cohort study. Using logistic regression and various machine learning modeling methods, we constructed multiple models to explore whether they could predict the malignancy and staging of gastrointestinal tumors, evaluate whether TEG five items are better at predicting gastrointestinal tumor malignancy compared to the Thromboelastogram Nine Items, and explore new applications of TEG and other coagulation indicators in gastrointestinal malignancies.

## 2 Methods

### 2.1 Patient information collection

We selected 300 patients with gastrointestinal diseases who were treated at the Second Affiliated Hospital of Anhui Medical University from January 2024 to August 2024. Their pathological and clinical data were retrospectively collected. The study was approved by the Ethics Committee of the Second Affiliated Hospital of Anhui Medical University (YX2023-183). Inclusion criteria were as follows: (1) Healthy group patients: No gastrointestinal tumors were diagnosed within one year after endoscopic examination; (2) Patients with benign gastrointestinal tumors: Benign gastrointestinal tumors were diagnosed by endoscopy and confirmed by pathology after resection; (3) Patients with malignant gastrointestinal tumors: Malignant gastrointestinal tumors were diagnosed by endoscopy and confirmed by pathology after resection. Exclusion criteria were: (1) Patients with tumors in other parts of the body; (2) Patients with other coagulation disorders or those who received anticoagulant therapy within three months; (3) Patients who received neoadjuvant chemotherapy before surgery; (4) Patients with acute inflammatory diseases.

Data were collected using the hospital’s case system, with data extracted from the patient’s electronic medical records, including laboratory indicators, tumor-related conditions, and basic information. Laboratory indicators included TEG, Thromboelastogram, tumor four items, three blood cell counts, and albumin and globulin. Tumor-related conditions included tumor size, tumor staging, tumor type, lymph node metastasis, vascular tumor thrombus, nerve invasion, TNM staging, G staging, and endoscopic findings. Essential information included gender, age, BMI, underlying diseases, and stool conditions. For comparative analysis, TNM and G stages were divided into two groups (I+II, III+IV; G1+G2, G3+G4).

### 2.2 Data analysis

Statistical analysis was performed using SPSS software (IBM, 26.0, USA) and GraphPad Prism software (GraphPad, 8.0, USA). Kruskal-Wallis test, Chi-square test, Mann-Whitney U test, and paired-sample t-test were used to compare differences between groups. Bonferroni correction was applied for pairwise comparisons. A one-way test was used to assess the unidirectional trend of TEG parameters. The Youden index was used as the optimal cutoff value, and receiver operating characteristic (ROC) analysis was performed to evaluate the potential of TEG parameters in distinguishing between benign and malignant gastrointestinal tumors. A P-value < 0.05 was considered statistically significant (two-sided). Next, stepwise multivariate logistic regression analysis was used to identify independent influencing factors for the malignancy of gastrointestinal tumors. A ROC curve was constructed to assess the predictive value of TEG-related indicators for the malignancy and staging of gastrointestinal tumors, and the area under the curve (AUC) and associated 95% confidence intervals (CI) were derived. Finally, a network model structure suitable for our task data was constructed based on residual networks. Our model incorporates residual structures, hence named the Residual Fully Connected Binary Classifier (RFCBC). To select the optimal model, this model was compared with other commonly used binary classification methods, such as logistic regression, support vector machines, decision trees, random forests, K-nearest neighbors, and naive Bayes.

## 3 Results

### 3.1 General information

**Figure 1:**
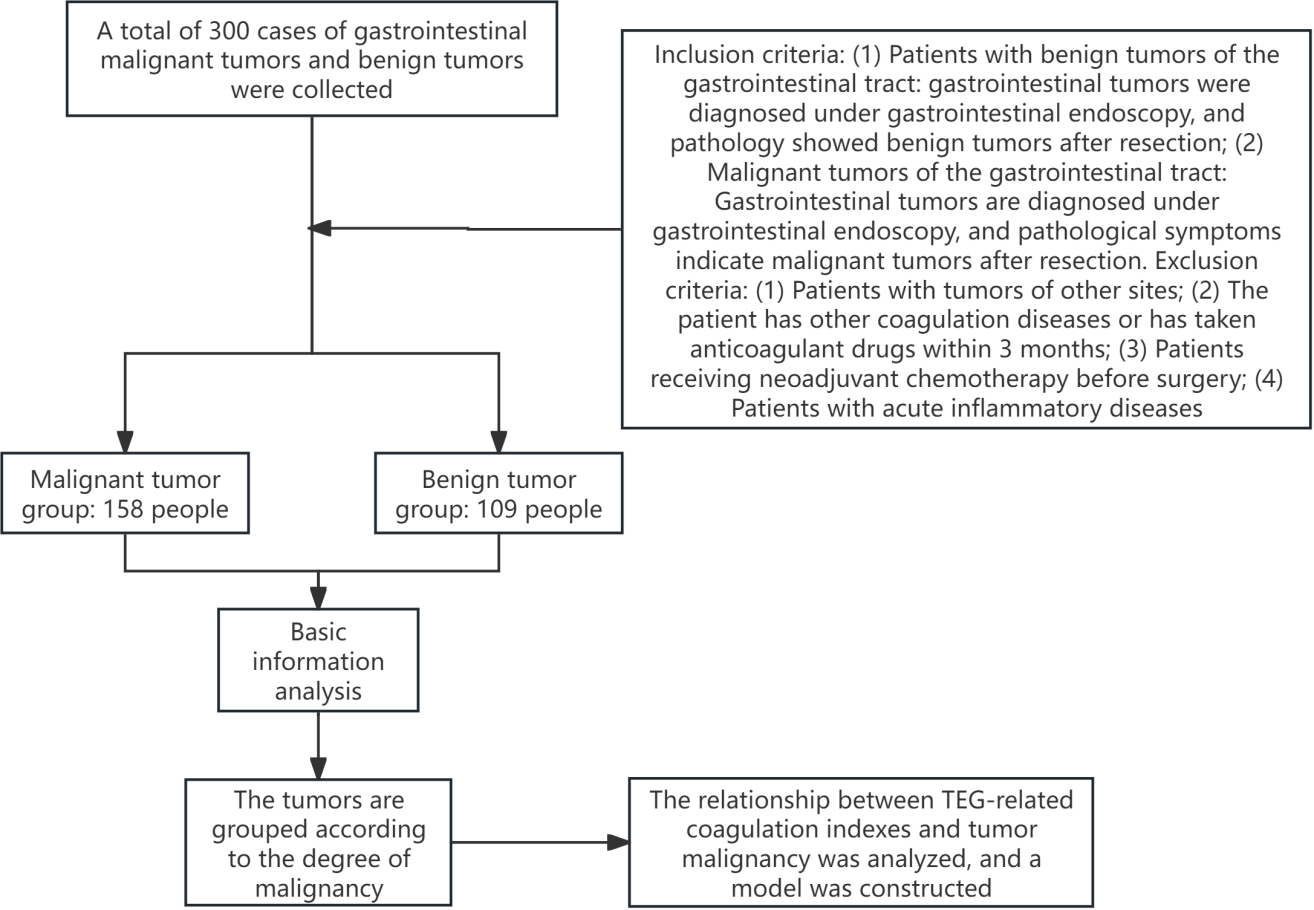
Flow chart of the study.

Finally, two hundred sixty-seven cases were included (**Figure 1**, excluding 10 cases with tumors in other parts of the body, 13 cases with neoadjuvant therapy, and 10 cases on anticoagulant drugs). Among them, 158 were malignant gastrointestinal tumors, and 109 were benign gastrointestinal tumors, all confirmed by pathology. The main demographic and clinical characteristics of the included patients are shown in **Table 1**. In short, the malignant tumor group had higher levels of age, smoking, male proportion, R, K, PT, INR, and FDP, and lower levels of angle, MA, CI, PTA, D-dimer, albumin, red blood cells, and hemoglobin (P < 0.05).

**Table 1:** Clinical participant baseline characteristics.

Variable	Malignant Tumor Group	Benign tumor group	*P*	95% CI
BMI	22.042 ± 4.451	22.650 ± 7.083	0.399	-1.999~0.783
Age	66.418 ± 10.872	55.458 ± 15.794	<0.001	7.747~14.171
Male	109	49	0.009	
Female	58	51		
Smoking				
Yes	62	96	0.005	
No	25	84		
Drinking				
Yes	56	102	0.601	
No	35	74		
Blood in the stool				
Yes	29	127	0.031	
No	9	96		
Hypertension				
Yes	77	81	0.211	
No	44	65		
TEG				
R	4.309 ± 0.952	4.828 ± 1.126	<0.001	-0.770 ~-0.267
K	1.518 ± 0.529	1.815 ± 0.665	<0.001	-0.448~-0.146
Angle	68.434 ± 6.839	65.116 ± 6.964	<0.001	1.623~5.015
MA	60.379 ± 6.946	58.891 ± 5.811	0.034	-0.057~3.032
CI	1.420 ± 1.753	0.494 ± 1.775	<0.001	0.493~1.360
Thrombostatic hemostasis				
PT	11.263 ± 1.577	11.027 ± 0.795	<0.001	-0.053~0.525
INR	0.933 ± 0.157	0.915 ± 0.085	0.003	-0.011~0.047
PTA	102.390 ± 20.164	110.411 ± 16.717	0.001	-12.485~-3.558
APTT	26.778 ± 5.139	27.251 ± 2.876	0.420	-1.444~0.498
FIB	8.528 ± 6.940	7.930 ± 6.977	0.489	-1.110 ~2.306
TT	11.363 ± 6.941	13.109 ± 11.339	0.405	-4.151~0.660
D-dimer	1.156 ± 2.044	1.173 ± 2.902	<0.001	-0.653~0.617
FDP	3.437 ± 6.362	3.429 ± 7.298	0.001	-1.693~1.709
AT-III	83.578 ± 18.685	87.762 ± 11.534	0.131	-8.144~-0.225
Other laboratory parameters				
Albumin	36.914 ± 4.495	39.919 ± 4.613	<0.001	-4.120 ~-1.891
Globulin	25.832 ± 5.325	25.295 ± 3.821	0.635	-0.632~1.706
Platelet	211.581 ± 85.362	205.642 ± 61.376	0.844	-12.816~24.694
White blood cell	6.973 ± 5.308	6.281 ± 2.440	0.307	-0.259~1.643
Neutrophils	5.107 ± 7.424	4.600 ± 6.703	0.136	-1.243~2.257
Lymphocyte	1.726 ± 2.218	1.610 ± 0.724	0.345	-0.317~0.550
Erythrocyte	3.872 ± 0.696	4.290 ± 0.700	<0.001	-0.589~-0.247
Hemoglobin	111.342 ± 25.016	127.982 ± 20.977	<0.001	-22.390 ~-10.890

**Figure 2:**
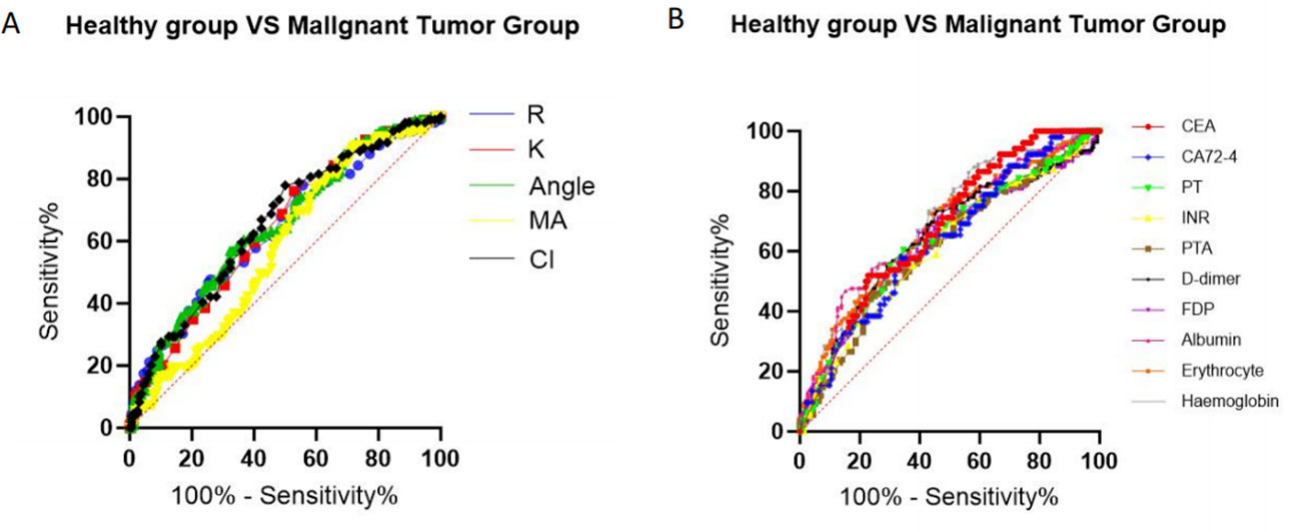
ROC curve analysis of TEG parameters and laboratory parameters for distinguishing the malignant tumor group from the benign tumor group (after propensity score matching). **(A)** TEG parameters. **(B)** Laboratory parameters.

The AUC values for R, K, α-angle, MA, and CI were 0.639, 0.644, 0.651, 0.576, and 0.658, respectively (P < 0.05). A comparison of the areas under the ROC curves indicated that TEG parameters were not proven to be superior to traditional laboratory indicators in distinguishing between benign and malignant gastrointestinal tumors (**Figure 2**, **Table 2**).

**Table 2:** ROC analysis of TEG and laboratory markers to distinguish between benign and malignant gastrointestinal tumors.

Variable	AUC	P	95%CI
TEG			
R	0.639	<0.001	0.571~0.707
K	0.644	<0.001	0.578~0.710
A	0.651	<0.001	0.584~0.717
MA	0.576	0.034	0.508~0.645
CI	0.658	<0.001	0.592~0.724
Laboratory parameters			
PT	0.638	<0.001	0.569~0.706
INR	0.613	0.002	0.544~0.682
PTA	0.613	0.002	0.544~0.682
D-dimer	0.649	<0.001	0.580~0.717
FDP	0.632	<0.001	0.562~0.702
Albumin	0.684	<0.001	0.619~0.749
Erythrocyte	0.671	<0.001	0.605~0.737
Hemoglobin	0.698	<0.001	0.635~0.761
CEA	0.679	<0.001	0.599~0.759
CA72-4	0.638	0.003	0.552~0.724

### 3.2 Influencing factors of TNM and G staging

This study grouped patients with malignant gastrointestinal tumors based on pathological results into TNM stages (I+II: 84 people, III+IV: 74 people) and G stages (G1+G2: 91 people, G3+G4: 67 people). Coagulation-related indicators were compared between the groups (**Table 3**). We found that the levels of the five TEG indicators differed significantly between the TNM and G stages. The FIB level in the I+II group was lower than in the III+IV group (7.468 ± 6.703 vs. 9.710 ± 7.033, P=0.043), while the APTT level in the G1+G2 group was higher than in the G3+G4 group (27.573 ± 5.762 vs. 25.690 ± 3.934, P=0.016). No significant differences were observed in other coagulation indicators in this study.

**Table 3:** Differences in coagulation-related markers under TNM and G stages.

Variable	TNM grouping		95%CI	*P*	G grouping		95%CI	*P*
	I+II	III+IV			G1+G2	G3+G4		
R	4.480 ± 0.927	4.114 ± 0.948	0.071~0.662	0.016	4.537 ± 0.953	3.985 ± 0.863	0.265~0.839	<0.001
K	1.660 ± 0.508	1.351 ± 0.508	0.148~0.468	<0.001	1.692 ± 0.553	1.282 ± 0.390	0.254~0.566	<0.001
Angle	67.157 ± 5.525	70.628 ± 5.349	-5.182 ~-1.760	<0.001	66.841 ± 5.884	71.357 ± 4.195	-6.181~-2.851	<0.001
MA	59.471 ± 6.165	62.327 ± 5.953	-4.762~-0.949	0.004	58.887 ± 6.046	63.054 ± 5.649	-6.019~-2.315	<0.001
CI	1.058 ± 1.535	2.026 ± 1.479	-1.441~-0.493	<0.001	0.923 ± 1.513	2.267 ± 1.319	-1.791~-0.897	<0.001
PT	11.130 ± 1.506	11.410 ± 1.650	-0.779~0.220	0.270	11.158 ± 1.505	11.388 ± 1.679	-0.742~0.282	0.376
INR	0.926 ± 0.135	0.942 ± 0.179	-0.067~0.034	0.517	0.929 ± 0.136	0.937 ± 0.184	0.045~-0.061	0.765
PTA	104.818 ± 19.649	99.850 ± 20.483	-1.363 ~11.298	0.123	104.763 ± 19.949	99.490 ± 20.351	-1.150~11.696	0.107
APTT	26.876 ± 3.952	26.670 ± 6.244	-1.466~1.877	0.808	27.573 ± 5.762	25.690 ± 3.934	0.358~3.408	0.016
FIB	7.468 ± 6.703	9.710 ± 7.033	-4.409~-0.074	0.043	8.007 ± 6.893	9.228 ± 6.999	-3.434~0.993	0.277
TT	12.306 ± 6.808	10.309 ± 6.964	-0.174~4.167	0.071	11.822 ± 7.031	10.747 ± 6.829	-1.126~3.275	0.336
D-dimer	0.896 ± 1.488	1.518 ± 2.584	-1.275~0.032	0.062	1.128 ± 2.287	1.192 ± 1.676	-0.686~0.559	0.841
FDP	3.130 ± 6.119	4.028 ± 6.934	-2.966 ~1.170	0.392	2.949 ± 4.585	4.090 ± 8.180	-3.343~1.059	0.306
AT-III	83.229 ± 20.420	83.970 ± 16.625	-6.568~5.085	0.802	84.126 ± 17.704	82.785 ± 20.038	-4.735~7.418	0.663

**Figure 3:**
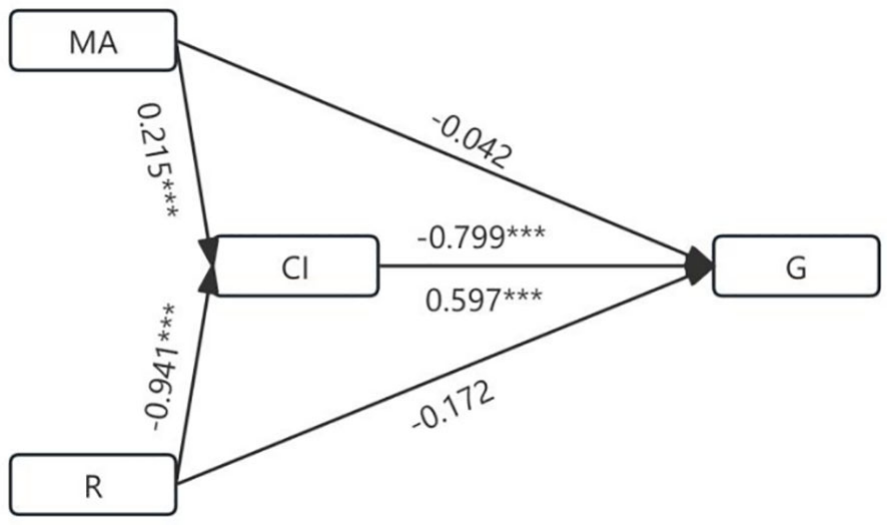
Diagram of the mediating effect model under stage G. ***P < 0.001.

**Figure 4:**
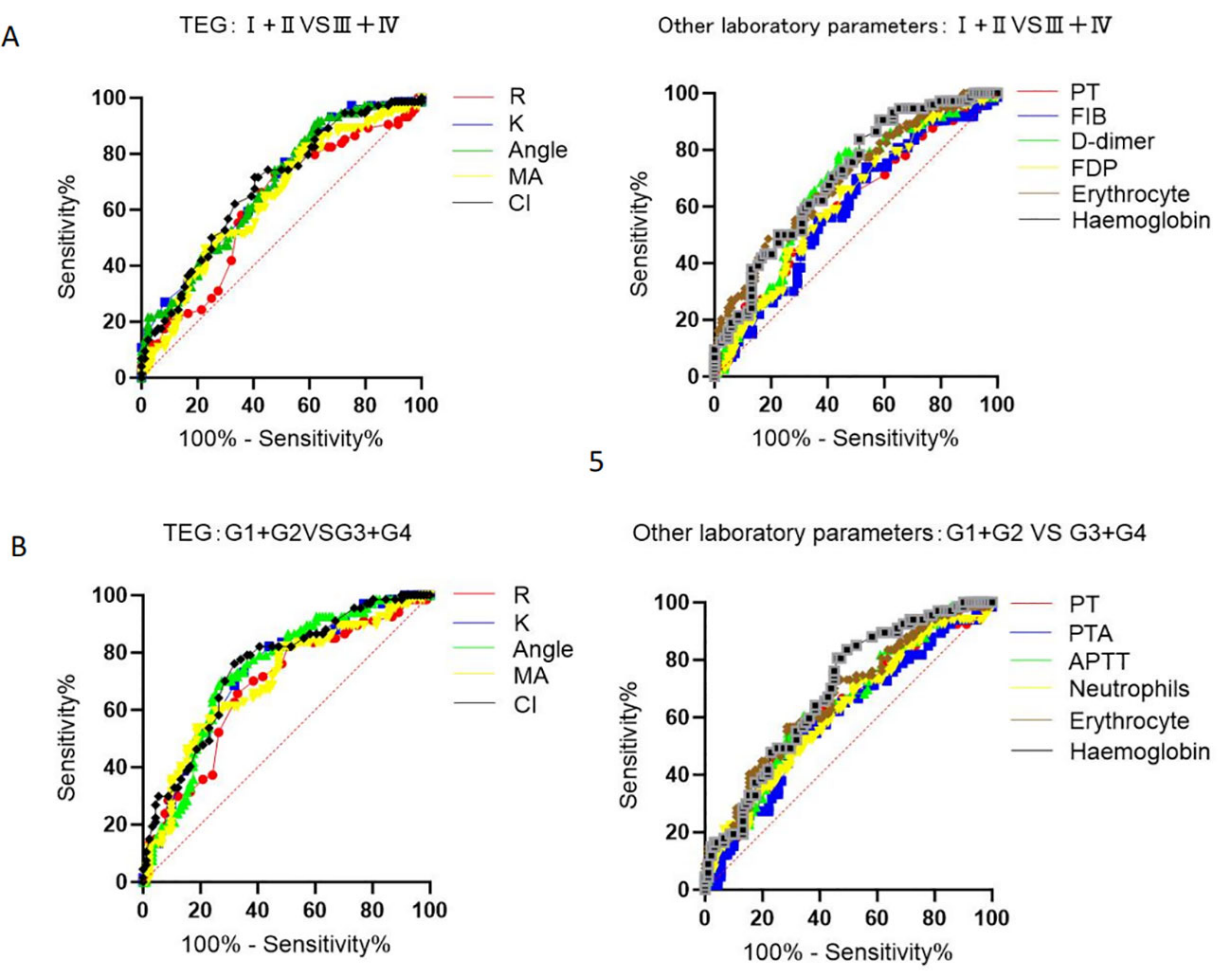
ROC curve analysis of TEG and laboratory parameters to distinguish between TNM and G groups (after propensity score matching). **(A)** TNM. **(B)** G.

Next, logistic regression analysis was conducted on the TNM and G groups. First, collinearity analysis was performed on the indicators with significance in univariate analysis and indicators with strong collinearity were excluded (**Supplementary Table S1**: K, α-angle, and CI values were excluded from TNM staging; K and α-angle values were excluded from G staging). Previous studies have shown that CI value is a composite indicator of R, K, α-angle, and MA values, so the possibility of a mediating effect should be considered before conducting multivariate regression. After constructing the mediation effect model, we found that the CI value was a complete mediator of the MA value and R-value in the G stage (**Figure 3**, **Table 4**). Therefore, the mediator variable CI was excluded during multivariate regression. Binary logistic regression analysis finally showed that R and MA values are independent influencing factors in both TNM and G groups (**Table 5**). Further ROC curve analysis of the TNM and G groups showed that the five TEG indicators (AUC values: R: 0.682; K: 0.731; α-angle: 0.736; MA: 0.699; CI: 0.747) had better discrimination ability in the G group compared to other coagulation indicators. However, although the TNM group showed moderate discrimination ability, they did not exhibit a significant advantage over other indicators (**Figure 4**, **Table 6**).

**Table 4:** Effect size and proportion of the mediating effect.

Effect relationship	Effect size	95%CI	Effect percentage
MA			
Total effect	0.121	0.062~0.181	
Direct effects	-0.0415	-0.149~0.066	-0.343
Indirect effects	0.172	0.084~0.301	1.421
R			
Total effect	-0.701	-1.099~-0.202	
Direct effects	-0.172	-0.634~0.290	0.245
Indirect effects	-0.561	-0.991~-0.292	0.800

**Table 5:** Univariate and multivariate analysis of laboratory indexes in different groups.

Variable	TNM						G					
	Univariate			Multivariate			Univariate			Multivariate		
	B	95%CI	*P*	B	95%CI	*P*	B	95%CI	*P*	B	95%CI	*P*
TEG												
R	0.650	0.455~0.929	0.018	0.654	0.451~0.949	0.025	0.496	0.333~0.738	0.001	0.633	0.415~0.967	0.034
K	0.267	0.127~0.560	<0.001				0.131	0.055~0.316	<0.001			
Angle	1.130	1.059~1.207	<0.001				1.196	1.108~1.290	<0.001			
MA	1.081	1.024~1.141	0.005	1.069	1.012~1.130	0.017	1.129	1.063~1.198	<0.001	1.092	1.092~1.027	0.005
CI	1.531	1.222~1.918	<0.001				1.925	1.483~2.500	<0.001			
Thrombostatic hemostasis												
PT	1.137	0.899~1.435	0.285				1.384	0.973~1.969	0.071			
INR	2.013	0.249~16.257	0.512				2.462	0.14~42.361	0.535			
PTA	0.987	0.971~1.004	0.129				0.982	0.963~1.001	0.070			
APTT	0.992	0.933~1.055	0.802				0.799	0.695~0.919	0.002	0.890	0.763~1.039	0.141
FIB	1.049	1.001~1.098	0.043	1.046	0.996~1.097	0.070	1.030	0.984~1.079	0.208			
TT	0.959	0.916~1.004	0.071				0.978	0.934~1.024	0.348			
D-dime	1.200	0.974~1.477	0.086				1.021	0.875~1.191	0.794			
FDP	1.022	0.971~1.076	0.398				1.030	0.976~1.087	0.280			
AT-III	1.002	0.985~1.019	0.803				1.001	0.980~1.023	0.970			
Other laboratory parameters												
Albumin	0.953	0.888~1.023	0.184				0.936	0.871~1.007	0.075			
Globulin	1.054	0.992~1.119	0.090				1.011	0.952~1.073	0.728			
Platelet	1.002	0.998~1.006	0.301				0.997	0.993~1.001	0.183			
White blood cell	0.995	0.937~1.056	0.874				1.004	0.947~1.065	0.889			
Neutrophils	1.090	0.960~1.236	0.183				1.175	1.019~1.356	0.027			
Lymphocyte	1.075	0.911~1.269	0.392				1.119	0.921~1.360	0.258			

**Table 6:** ROC analysis of TEGs and laboratory markers was performed to distinguish the degree of malignancy of gastrointestinal tumors.

Variable	TNM			G		
	AUC	P	95%CI	AUC	P	95%CI
TEG						
R	0.615	0.013	0.526~0.703	0.682	<0.001	0.598~0.766
K	0.682	<0.001	0.600~0.764	0.731	<0.001	0.653~0.810
A	0.680	<0.001	0.598~0.763	0.736	<0.001	0.658~0.814
MA	0.637	<0.001	0.550~0.723	0.699	<0.001	0.616~0.783
CI	0.683	<0.001	0.600~0.765	0.747	<0.001	0.671~0.824
Other laboratory parameters						
PT	0.603	0.026	0.515~0.692	0.618	0.012	0.529~0.708
FIB	0.595	0.043	0.505~0.684	0.597	0.039	0.507~0.687
D-dime	0.665	<0.001	0.579~0.751	0.648	<0.001	0.561~0.734
FDP	0.619	0.010	0.532~0.707	0.619	0.011	0.531~0.708
Erythrocyte	0.695	<0.001	0.614~0.777	0.667	<0.001	0.582~0.752
Hemoglobin	0.705	<0.001	0.625~0.785	0.692	<0.001	0.610~0.773

### 3.3 Influencing factors of R and MA values

**Figure 5:**
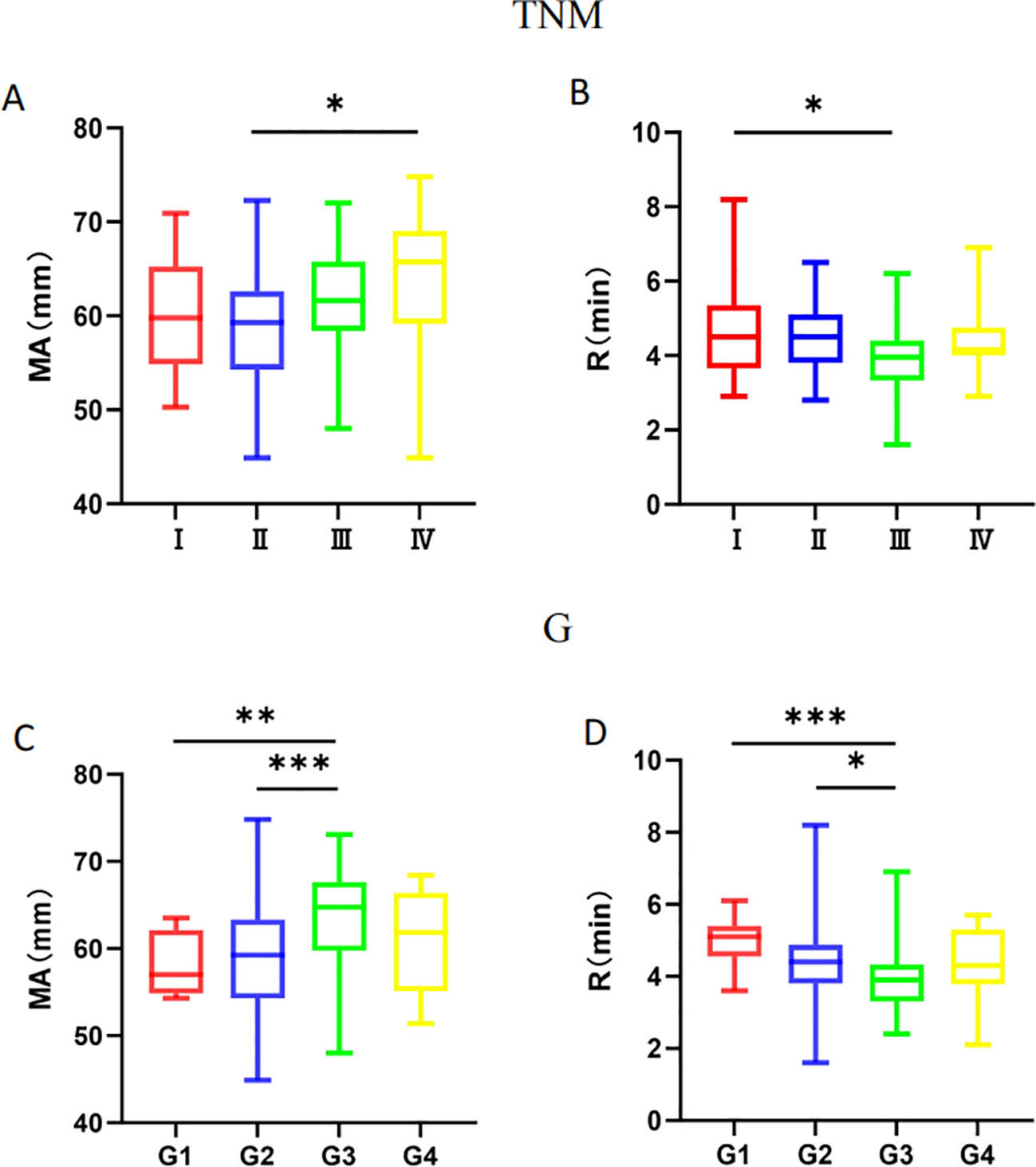
Comparison of the difference between MA value and R-value in TNM group and G group. **(A)** There was a difference in the expression of MA values between II. and IV. in the TNM group. **(B)** There was a difference in the expression of the R-value between I and III. in the TNM group. **(C)** There were differences in the expression of MA values between G1, G2, and G3 in the TNM group. **(D)** There were differences in the expression of R values between G1, G2, and G3 in the TNM group. * 0.01≤P<0.05, ** 0.001≤P<0.01, *** P<0.001.

The binary logistic regression analysis results indicated that R and MA values are independent influencing factors within TEG indicators for both TNM and G groups. We conducted a more detailed analysis by dividing the TNM and G stages into four groups to explore further. The analysis revealed that in the TNM stages, the R-value in stage I was higher than in stage III (P < 0.05), and the MA value in stage IV was higher than in stage II (P < 0.05). In the G stages, the R-value in stage G3 was lower than in stages G2 and G1 (P < 0.05), and the MA value in stage G3 was higher than in stages G2 and G1 (P < 0.05) (**Figure 5**).

Single-factor analysis was used to identify the influencing factors of R and MA values. Stepwise linear regression was then employed to determine the independent influencing factors of R and MA values. The independent influencing factors for R included hemoglobin (B: 0.254; CI: 0.003-0.016; P: 0.005), carbohydrate antigen 19-9 (B: 0.227; CI: 0.000-0.001; P: 0.003), and VTE (Caprini) score (B: -0.240; CI: -0.166 to -0.035; P: 0.003). The independent influencing factors for MA included globulin (B: 0.179; CI: 0.057-0.366; P: 0.008), platelets (B: 0.374; CI: 0.017-0.036; P: <0.001), hemoglobin (B: -0.307; CI: -0.109 to -0.042; P: <0.001), and VTE (Caprini) score (B: 0.209; CI: 0.210-0.941; P: 0.002) (**Table 7**).

**Table 7:** Univariate and multivariate analysis of laboratory indexes in different groups.

Variable	R						MA					
	Univariate			Multivariate			Univariate			Multivariate		
	B	95%CI	*P*	B	95%CI	*P*	B	95%CI	*P*	B	95%CI	*P*
Thrombostatic hemostasis												
PT	0.026	-0.135~0.190	0.742				0.103	-0.363~1.750	0.196			
INR	0.065	-0.759~1.833	0.415				0.064	-5.048~11.875	0.427			
PTA	0.023	-0.008~0.010	0.772				-0.119	-0.104~0.014	0.137			
APTT	0.229	0.017~0.088	0.004	0.130	-0.005~0.063	0.093	-0.197	-0.528~-0.063	0.013	-0.077	-0.303~0.080	0.252
FIB	0.088	-0.010~0.034	0.274				0.105	-0.047~0.236	0.188			
TT	-0.109	-0.037~0.007	0.173				-0.089	-0.224~0.062	0.264			
D-dimer	0.011	-0.067~0.077	0.888				0.048	-0.329~0.612	0.553			
FDP	0.115	-0.006~0.040	0.148				0.144	-0.012~0.287	0.072			
AT-III	0.118	-0.002~0.017	0.141				0.096	-0.025~0.101	0.232			
Other laboratory parameters												
Albumin	0.281	0.027~0.092	<0.001	0.089	-0.019~0.057	0.329	-0.131	-0.398~0.036	0.102			
Globulin	0.093	-0.011~0.045	0.244				0.229	0.087~0.447	0.004	0.179	0.057~0.366	0.008
Platelet	0.045	-0.001~0.002	0.576				0.467	0.024~0.044	<0.001	0.374	0.017~0.036	<0.001
White blood cell	-0.052	-0.038~0.019	0.513				0.103	-0.063~0.305	0.197			
Neutrophils	-0.081	-0.031~0.010	0.310				0.072	-0.072~0.192	0.372			
Lymphocyte	-0.045	-0.087~0.049	0.578				-0.019	-0.495~0.390	0.815			
Erythrocyte	0.299	0.202~0.613	<0.001				-0.296	-3.976~-1.289	<0.001			
Hemoglobin	0.378	0.009~0.020	<0.001	0.254	0.003~0.016	0.005	-0.410	-0.136~-0.065	<0.001	-0.307	-0.109~-0.042	<0.001
Tumor-related indicators												
Tumor length	-0.047	-0.110~0.061	0.577				0.167	0.007~1.165	0.047	0.053	-0.272~0.646	0.421
Tumor area	0.002	-0.013~0.013	0.981				0.160	-0.003~0.173	0.058			
Alpha-fetoprotein	-0.119	0.000~0.000	0.159				0.060	0.000~0.001	0.479			
Carcinoembryonic antigen	0.124	-0.001~0.004	0.141				0.143	-0.002~0.025	0.090			
Carbohydrate antigens 19-9	0.173	0.000~0.001	0.040	0.227	0.000~0.001	0.003	0.135	-0.001~0.008	0.110			
CA72-4	0.149	0.000~0.008	0.077				0.154	-0.002~0.051	0.068			
General indicators												
Age	-0.204	-0.031~-0.004	0.010	-0.030	-0.019~0.013	0.715	0.045	-0.065~0.116	0.578			
BMI	0.065	-0.027-0.066	0.419				0.037	-0.232~0.372	0.647			
VTE (Caprini) score	-0.330	-0.201~-0.076	<0.001	-0.240	-0.166~-0.035	0.003	0.301	0.410~1.234	<0.001	0.209	0.210~0.941	0.002
Nutritional screening	-0.020	-0.128~0.099	0.805				0.087	-0.331~1.133	0.281			

### 3.4 Construction of prediction models for gastrointestinal tumor malignancy Using TEG and other coagulation indicators based on machine learning methods

**Figure 6:**
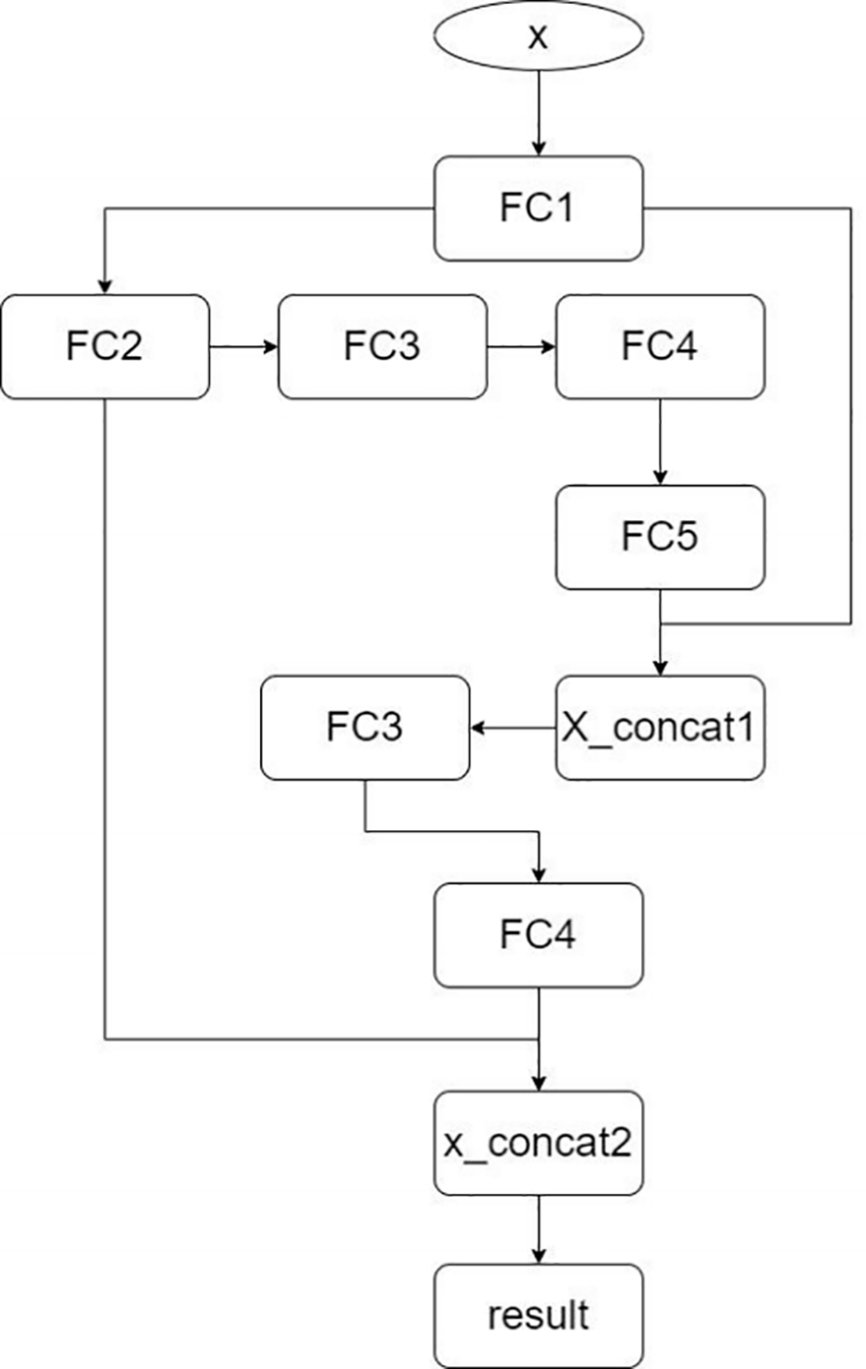
Model diagram of the residual fully connected binary classifier (RFCBC).

**Figure 7:**
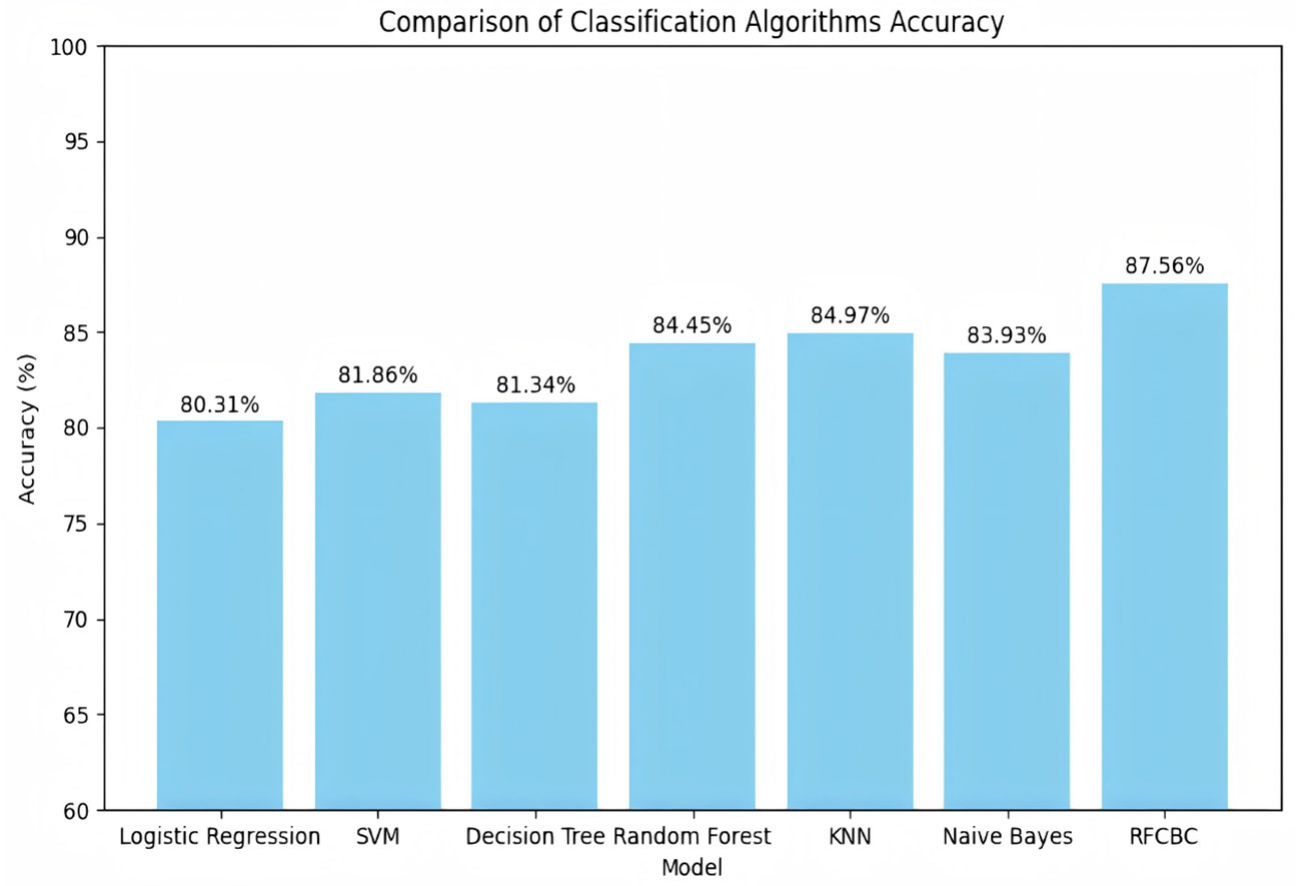
Inference results of multiple models for the five items of TEG data.

**Figure 8:**
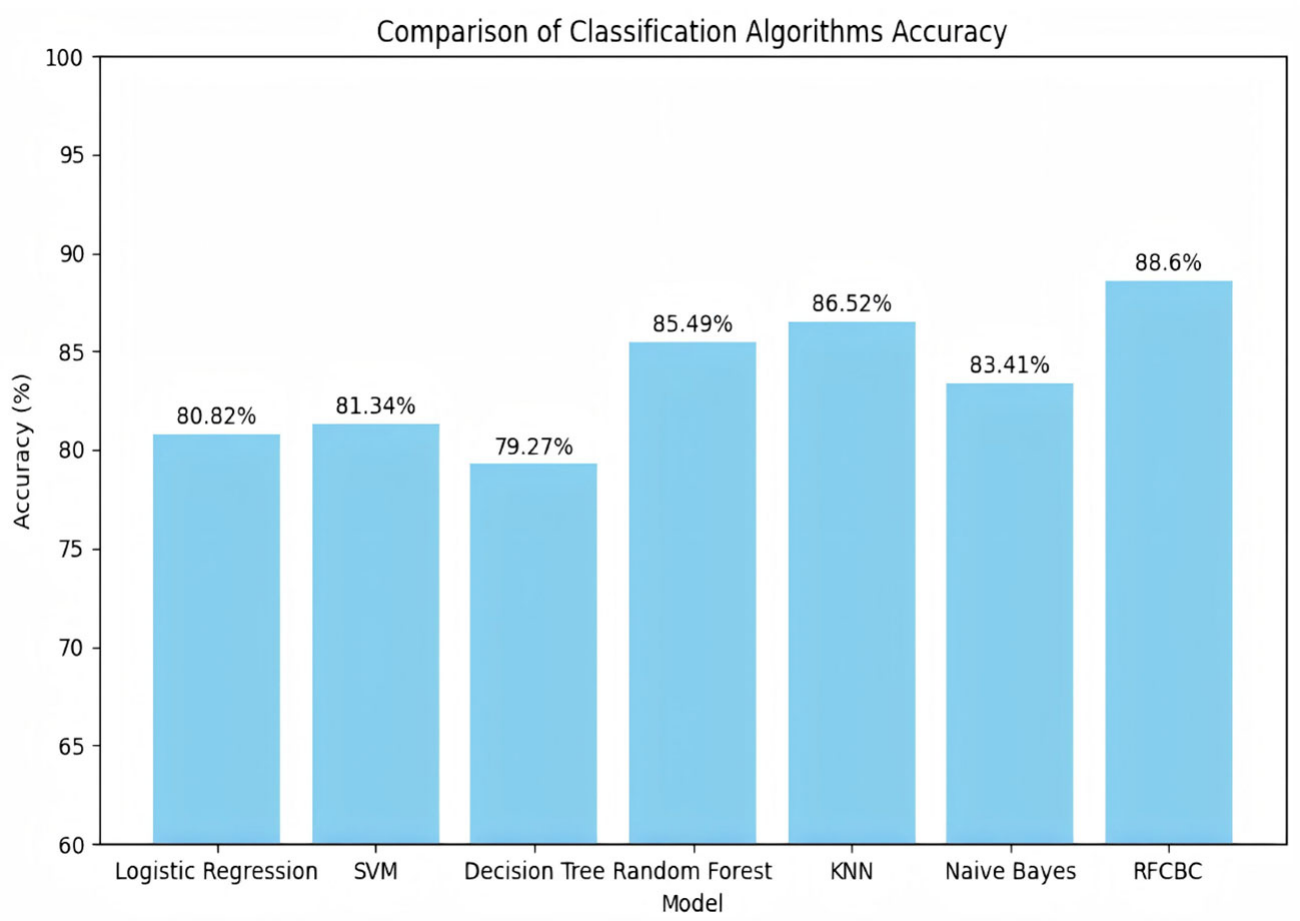
Nine data on thrombosis and inference results of various models.

The model structure follows: The input layer accepts the input vector, which passes through the first fully connected layer (FC1), expanding the feature dimension to 64. Then, the second (FC2), third (FC3), and fourth (FC4) fully connected layers progressively increase the feature dimension to 128, 256, and 512, respectively. The fifth fully connected layer (FC5) reduces the feature dimension to 256, allowing it to concatenate with the output of the first fully connected layer. In the intermediate stage of the model, the output of FC1 is first concatenated with the output of FC5 along the feature dimension to form a new feature vector. The concatenated feature vector is processed again through FC3 and FC4 to extract further and integrate features. Finally, the output of FC2 is concatenated with the reprocessed feature vector along the feature dimension for a second time. The concatenated feature vector is then passed through the last fully connected layer (FC6), which reduces the feature dimension from 512 to 2, corresponding to the two classification labels. The model structure is shown in **Figure 6**. For all samples, the model’s inference results for the TEG five-item data are shown in **Figure 7**, and for the Thromboelastogram Nine-Item data, the inference results are shown in **Figure 8**. The RFCBC prediction model performed better than other commonly used binary classification methods (TEG five items: 87.56%; Thromboelastogram Nine Items: 88.6%).

## 4 Discussion

Current research indicates that coagulation-related components mediate the progression of gastrointestinal malignancies (13). The incidence of coagulation-related diseases in cancer patients is higher than in those with benign diseases, with thrombotic events occurring in 4.5% of gastric cancer patients and 2.3% of colon cancer patients (14). Moreover, patients with advanced-stage, high-malignancy cancers, especially those with metastatic disease, are at a higher risk of developing coagulation-related diseases compared to patients with early-stage, low-malignancy cancers (15–17). These tumor cell-mediated hypercoagulable states further promote tumor cell proliferation and metastasis. Therefore, routine screening of hematological indicators such as Thromboelastogram Nine Items and blood counts is usually conducted preoperatively for patients with gastrointestinal malignancies to guide treatment (18, 19). Currently, TEG, with its advantages of being rapid and dynamic, is increasingly used for coagulation function detection in cancer. However, its additional advantages over traditional coagulation indicators in cancer are yet to be fully explored. Based on the above conclusions and considering the characteristic that gastrointestinal malignancies are generally detected at later stages, this study aims to explore the relationship between coagulation indicators such as TEG, Thromboelastogram Nine Items, blood counts, and common tumor markers with the staging and malignancy of gastrointestinal malignancies through various models constructed using machine learning and logistics regression. It also seeks to establish relevant prediction models to provide feasible methods and approaches for the future non-invasive preoperative prediction of gastrointestinal malignancies.

Initially, 300 patients were included in the study, with 33 patients excluded based on exclusion criteria. The remaining patients comprised 158 cases of malignant gastrointestinal tumors and 109 cases of benign gastrointestinal tumors. Due to the study’s retrospective nature and the need to confirm tumor staging and malignancy through postoperative pathology, relatively few patients were in the T1, T4, G1, and G4 stages. Therefore, TNM groups were divided into T1+T2 and T3+T4, and G groups were divided into G1+G2 and G3+G4 for comparison and analysis.

Previous studies have suggested that patients with malignant tumors exhibit a hypercoagulable state compared to healthy individuals (20), characterized by decreased R and K values and increased MA, α-angle, and CI values. Therefore, we first investigated the differences in TEG and other indicators between benign and malignant gastrointestinal tumors before exploring the relationship between coagulation indicators and gastrointestinal malignancies. Our analysis revealed that the differences in the five TEG indicators were consistent with previous studies, confirming that patients with gastrointestinal malignancies are in a hypercoagulable state. Moreover, among the Thromboelastogram Nine Items, differences in PT, INR, PTA, D-dimer, and FDP were observed, indicating that the coagulation factors and fibrinolysis systems in patients with gastrointestinal malignancies are in a higher state of activation (21), consistent with previous research results. Analysis of blood counts and general indicators also revealed lower albumin and hemoglobin levels in patients with gastrointestinal malignancies, likely related to difficulties in eating and blood in the stool. Further analysis indicated that R, K, α-angle, and CI could be potential indicators for distinguishing between benign and malignant gastrointestinal tumors. Unfortunately, however, they did not perform better than traditional indicators.

Given that previous studies have indicated that indicators like MA are associated with lymph node metastasis in gastric cancer patients (13), we became interested in whether TEG and other related coagulation indicators could be used to predict the malignancy of gastrointestinal tumors. We first used stepwise logistic regression analysis, excluding highly collinear indicators and mediator variables, and identified R and MA values as independent predictors in both TNM and G groups. The R-value represents the clot reaction time, primarily reflecting the combined effect of coagulation factors involved in coagulation initiation, indicating the coagulation factors’ activity. The MA value in TEG represents the maximum amplitude, which mainly reflects platelet aggregation function and the quality of fibrinogen (22).

Further analysis of R and MA values revealed that the R-value decreases as TNM and G stages progress, while the MA value increases with advancing stages. This indicates that as the malignancy of the tumor increases, the activity of coagulation factors, platelets, and fibrinogen also increases. Previous research has shown that activated platelets can significantly inhibit T-cell proliferation and NK cell activity through the GARP/TGF-β pathway (23). Similarly, the activation of fibrinogen also promotes tumor proliferation and metastasis (24). We included relevant tumor markers and performed stepwise linear regression to study the factors influencing R and MA values. We found that hemoglobin, carbohydrate antigen 19-9, and VTE scores are independent predictors of the R-value, while globulin, platelets, hemoglobin, and VTE scores are independent predictors of the MA value. This analysis revealed that R and MA values in coagulation indicators could effectively predict tumor malignancy, and there is a close relationship between TEG and tumor VTE scores.

To further explore the application value of TEG and other related coagulation indicators in distinguishing the malignancy of gastrointestinal tumors, we first used ROC curve analysis. We found that the TEG five items had better discrimination ability in the G group than other coagulation indicators. In contrast, in the TNM group, although the discrimination ability was moderate, it did not show a significant advantage over other indicators. This finding intrigued us, and to further compare the predictive power of the TEG five items with the Thromboelastogram Nine Items in G staging and construct a specific predictive model, we developed a relevant deep learning model. Given the limited number of variables in our data and the small sample size, using a network model structure that is too complex could lead to overfitting.

In contrast, a sparse network structure could result in poor convergence. Therefore, based on residual networks, we constructed a network model structure suitable for our task data. We incorporated residual structures into our model, naming it the Residual Fully Connected Binary Classifier (RFCBC). When compared with other commonly used binary classification methods, our model achieved the best experimental results (the prediction accuracy for the TEG five items and the Thromboelastogram Nine Items models were 87.56% and 88.6%, respectively), providing a reliable method for identifying whether a gastrointestinal tumor is malignant and assisting doctors in making effective auxiliary diagnoses. Additionally, based on our model, there was no significant difference in the predictive ability between the TEG five items and the Thromboelastogram Nine Items data.

In conclusion, this study found that R and MA values are independent predictors of gastrointestinal tumor malignancy among coagulation indicators. The TEG five items have better discrimination ability for G staging than other hematological indicators. Additionally, in experiments predicting gastrointestinal tumor malignancy, the RFCBC model created in this study outperformed other commonly used binary classification methods, such as logistic regression, support vector machines, decision trees, random forests, K-nearest neighbors, and naive Bayes, offering a new model construction method and feasible approach for future coagulation index predictions of gastrointestinal tumor malignancy. However, this study has limitations, such as a small sample size and a lack of multi-center data to validate the model. Future research should include more data to validate the results and model and incorporate more variables to conduct coagulation index-level predictions. Integrating machine learning methods into the prognosis and prediction of gastrointestinal malignancies will lead to a more comprehensive tumor treatment and prognosis prediction system.

## Data Availability

The original contributions presented in the study are included in the article/**Supplementary Material**. Further inquiries can be directed to the corresponding authors.
