# Supplementary material for: Prediction model of gastrointestinal tumor malignancy based on coagulation indicators such as TEG and neural networks
Source: Front Immunol. 2025 Mar 25;16:1507773. doi: 10.3389/fimmu.2025.1507773 (PMC11975555; doi:10.3389/fimmu.2025.1507773)
Supplement: Supplementary file 1 [file DataSheet1.docx]

| S-TABLE1：Comparison of AUC values of TEG and other coagulation indicators | | | | |
| --- | --- | --- | --- | --- |
| Variable | PT | FIB | D-dime | FDP |
| R | ＜0.001 | ＜0.001 | ＜0.001 | ＜0.001 |
| K | ＜0.001 | ＜0.001 | ＜0.001 | ＜0.001 |
| A | 0.051 | 0.011 | 0.017 | 0.005 |
| MA | 0.194 | 0.061 | 0.086 | 0.032 |
| CI | 0.032 | 0.006 | 0.010 | 0.002 |

| **Regional difference of independent variable group under ROC curve** | | | | | |
| --- | --- | --- | --- | --- | --- |
| Test result variable: testR-FIB | | | | | |
| asymptotic | | AUC discrepancy | Standard error difference^b^ | asymptotic 95% CI | |
| z | Sig.（Double tail）^a^ |  |  | floor | Upper limit |
| -4.198 | .000 | -.264 | .063 | -.387 | -.141 |
| a. Null hypothesis: true regiondiscrepancy = 0 | | | | | |
| b. Assumed as nonparametric | | | | | |

| **Regional difference of independent variable group under ROC curve** | | | | | |
| --- | --- | --- | --- | --- | --- |
| Test result variable: testR-PT | | | | | |
| asymptotic | | AUC discrepancy | Standard error difference^b^ | asymptotic 95% CI | |
| z | Sig.（Double tail）^a^ |  |  | floor | Upper limit |
| -4.778 | .000 | -.300 | .063 | -.423 | -.177 |
| a. Null hypothesis: true regiondiscrepancy = 0 | | | | | |
| b. Assumed as nonparametric | | | | | |

| **Regional difference of independent variable group under ROC curve** | | | | | |
| --- | --- | --- | --- | --- | --- |
| Test result variable: testR-D2 | | | | | |
| asymptotic | | AUC discrepancy | Standard error difference^b^ | asymptotic 95% CI | |
| z | Sig.（Double tail）^a^ |  |  | floor | Upper limit |
| -4.321 | .000 | -.273 | .063 | -.397 | -.149 |
| a. Null hypothesis: true regiondiscrepancy = 0 | | | | | |
| b. Assumed as nonparametric | | | | | |

| **Regional difference of independent variable group under ROC curve** | | | | | |
| --- | --- | --- | --- | --- | --- |
| Test result variable: testR-FDP | | | | | |
| asymptotic | | AUC discrepancy | Standard error difference^b^ | asymptotic 95% CI | |
| z | Sig.（Double tail）^a^ |  |  | floor | Upper limit |
| -3.903 | .000 | -.246 | .063 | -.370 | -.123 |
| a. Null hypothesis: true regiondiscrepancy = 0 | | | | | |
| b. Assumed as nonparametric | | | | | |

| **Regional difference of independent variable group under ROC curve** | | | | | |
| --- | --- | --- | --- | --- | --- |
| Test result variable: testK-FIB | | | | | |
| asymptotic | | AUC discrepancy | Standard error difference^b^ | asymptotic 95% CI | |
| z | Sig.（Double tail）^a^ |  |  | floor | Upper limit |
| -5.154 | .000 | -.313 | .061 | -.433 | -.194 |
| a. Null hypothesis: true regiondiscrepancy = 0 | | | | | |
| b. Assumed as nonparametric | | | | | |

| **Regional difference of independent variable group under ROC curve** | | | | | |
| --- | --- | --- | --- | --- | --- |
| Test result variable: testK-PT | | | | | |
| asymptotic | | AUC discrepancy | Standard error difference^b^ | asymptotic 95% CI | |
| z | Sig.（Double tail）^a^ |  |  | floor | Upper limit |
| -5.755 | .000 | -.350 | .061 | -.469 | -.231 |
| a. Null hypothesis: true regiondiscrepancy = 0 | | | | | |
| b. Assumed as nonparametric | | | | | |

| **Regional difference of independent variable group under ROC curve** | | | | | |
| --- | --- | --- | --- | --- | --- |
| Test result variable: tsstK-D2 | | | | | |
| asymptotic | | AUC discrepancy | Standard error difference^b^ | asymptotic 95% CI | |
| z | Sig.（Double tail）^a^ |  |  | floor | Upper limit |
| -5.275 | .000 | -.323 | .061 | -.442 | -.203 |
| a. Null hypothesis: true regiondiscrepancy = 0 | | | | | |
| b. Assumed as nonparametric | | | | | |

| **Regional difference of independent variable group under ROC curve** | | | | | |
| --- | --- | --- | --- | --- | --- |
| Test result variable: testK-FDP | | | | | |
| asymptotic | | AUC discrepancy | Standard error difference^b^ | asymptotic 95% CI | |
| z | Sig.（Double tail）^a^ |  |  | floor | Upper limit |
| -4.844 | .000 | -.296 | .061 | -.416 | -.176 |
| a. Null hypothesis: true regiondiscrepancy = 0 | | | | | |
| b. Assumed as nonparametric | | | | | |

| **Regional difference of independent variable group under ROC curve** | | | | | |
| --- | --- | --- | --- | --- | --- |
| Test result variable: testA-FIB | | | | | |
| asymptotic | | AUC discrepancy | Standard error difference^b^ | asymptotic 95% CI | |
| z | Sig.（Double tail）^a^ |  |  | floor | Upper limit |
| 2.541 | .011 | .154 | .061 | .035 | .273 |
| a. Null hypothesis: true regiondiscrepancy = 0 | | | | | |
| b. Assumed as nonparametric | | | | | |

| **Regional difference of independent variable group under ROC curve** | | | | | |
| --- | --- | --- | --- | --- | --- |
| Test result variable: testA-PT | | | | | |
| asymptotic | | AUC discrepancy | Standard error difference^b^ | asymptotic 95% CI | |
| z | Sig.（Double tail）^a^ |  |  | floor | Upper limit |
| 1.948 | .051 | .118 | .061 | -.001 | .237 |
| a. Null hypothesis: true regiondiscrepancy = 0 | | | | | |
| b. Assumed as nonparametric | | | | | |

| **Regional difference of independent variable group under ROC curve** | | | | | |
| --- | --- | --- | --- | --- | --- |
| Test result variable: testA-D2 | | | | | |
| asymptotic | | AUC discrepancy | Standard error difference^b^ | asymptotic 95% CI | |
| z | Sig.（Double tail）^a^ |  |  | floor | Upper limit |
| 2.378 | .017 | .145 | .061 | .025 | .265 |
| a. Null hypothesis: true regiondiscrepancy = 0 | | | | | |
| b. Assumed as nonparametric | | | | | |

| **Regional difference of independent variable group under ROC curve** | | | | | |
| --- | --- | --- | --- | --- | --- |
| Test result variable: testA-FDP | | | | | |
| asymptotic | | AUC discrepancy | Standard error difference^b^ | asymptotic 95% CI | |
| z | Sig.（Double tail）^a^ |  |  | floor | Upper limit |
| 2.821 | .005 | .172 | .061 | .052 | .291 |
| a. Null hypothesis: true regiondiscrepancy = 0 | | | | | |
| b. Assumed as nonparametric | | | | | |

| **Regional difference of independent variable group under ROC curve** | | | | | |
| --- | --- | --- | --- | --- | --- |
| Test result variable: testMA-FIB | | | | | |
| asymptotic | | AUC discrepancy | Standard error difference^b^ | asymptotic 95% CI | |
| z | Sig.（Double tail）^a^ |  |  | floor | Upper limit |
| 1.875 | .061 | .117 | .063 | -.005 | .240 |
| a. Null hypothesis: true regiondiscrepancy = 0 | | | | | |
| b. Assumed as nonparametric | | | | | |

| **Regional difference of independent variable group under ROC curve** | | | | | |
| --- | --- | --- | --- | --- | --- |
| Test result variable: testMA-PT | | | | | |
| asymptotic | | AUC discrepancy | Standard error difference^b^ | asymptotic 95% CI | |
| z | Sig.（Double tail）^a^ |  |  | floor | Upper limit |
| 1.298 | .194 | .081 | .062 | -.041 | .204 |
| a. Null hypothesis: true regiondiscrepancy = 0 | | | | | |
| b. Assumed as nonparametric | | | | | |

| **Regional difference of independent variable group under ROC curve** | | | | | |
| --- | --- | --- | --- | --- | --- |
| Test result variable: testMA-D2 | | | | | |
| asymptotic | | AUC discrepancy | Standard error difference^b^ | asymptotic 95% CI | |
| z | Sig.（Double tail）^a^ |  |  | floor | Upper limit |
| 1.720 | .086 | .108 | .063 | -.015 | .231 |
| a. Null hypothesis: true regiondiscrepancy = 0 | | | | | |
| b. Assumed as nonparametric | | | | | |

| **Regional difference of independent variable group under ROC curve** | | | | | |
| --- | --- | --- | --- | --- | --- |
| Test result variable: testMA-FDP | | | | | |
| asymptotic | | AUC discrepancy | Standard error difference^b^ | asymptotic 95% CI | |
| z | Sig.（Double tail）^a^ |  |  | floor | Upper limit |
| 2.149 | .032 | .135 | .063 | .012 | .258 |
| a. Null hypothesis: true regiondiscrepancy = 0 | | | | | |
| b. Assumed as nonparametric | | | | | |

| **Regional difference of independent variable group under ROC curve** | | | | | |
| --- | --- | --- | --- | --- | --- |
| Test result variable: testCI-FIB | | | | | |
| asymptotic | | AUC discrepancy | Standard error difference^b^ | asymptotic 95% CI | |
| z | Sig.（Double tail）^a^ |  |  | floor | Upper limit |
| 2.746 | .006 | .165 | .060 | .047 | .283 |
| a. Null hypothesis: true regiondiscrepancy = 0 | | | | | |
| b. Assumed as nonparametric | | | | | |

| **Regional difference of independent variable group under ROC curve** | | | | | |
| --- | --- | --- | --- | --- | --- |
| Test result variable: testCI-PT | | | | | |
| asymptotic | | AUC discrepancy | Standard error difference^b^ | asymptotic 95% CI | |
| z | Sig.（Double tail）^a^ |  |  | floor | Upper limit |
| 2.149 | .032 | .129 | .060 | .011 | .247 |
| a. Null hypothesis: true regiondiscrepancy = 0 | | | | | |
| b. Assumed as nonparametric | | | | | |

| **Regional difference of independent variable group under ROC curve** | | | | | |
| --- | --- | --- | --- | --- | --- |
| Test result variable: testCI-D2 | | | | | |
| asymptotic | | AUC discrepancy | Standard error difference^b^ | asymptotic 95% CI | |
| z | Sig.（Double tail）^a^ |  |  | floor | Upper limit |
| 2.580 | .010 | .156 | .061 | .038 | .275 |
| a. Null hypothesis: true regiondiscrepancy = 0 | | | | | |
| b. Assumed as nonparametric | | | | | |

| **Regional difference of independent variable group under ROC curve** | | | | | |
| --- | --- | --- | --- | --- | --- |
| Test result variable: testCI-FDP | | | | | |
| asymptotic | | AUC discrepancy | Standard error difference^b^ | asymptotic 95% CI | |
| z | Sig.（Double tail）^a^ |  |  | floor | Upper limit |
| 3.027 | .002 | .183 | .060 | .065 | .301 |
| a. Null hypothesis: true regiondiscrepancy = 0 | | | | | |
| b. Assumed as nonparametric | | | | | |
