# Supplementary material for: Prediction model of gastrointestinal tumor malignancy based on coagulation indicators such as TEG and neural networks
Source: Front Immunol. 2025 Mar 25;16:1507773. doi: 10.3389/fimmu.2025.1507773 (PMC11975555; doi:10.3389/fimmu.2025.1507773)
Supplement: Supplementary file 2 [file Table1.docx]

FIG S1: Collinearity analysis

| TNM |  |  |  |  |
| --- | --- | --- | --- | --- |
|  | β | t | *P* | VIF |
| （constant） | 0.076 | 0 | 1 |  |
| Zscore(K) | 0.266 | -0.289 | 0.773 | 12.04 |
| Zscore(A) | 0.297 | 0.243 | 0.808 | 14.98 |
| Zscore(CI) | 0.244 | 1.113 | 0.267 | 10.106 |
| Zscore(MA) | 0.163 | -0.722 | 0.471 | 4.519 |
|  |  |  |  |  |
|  |  |  |  |  |
| G分组 |  |  |  |  |
|  | β | t | *P* | VIF |
| （constant） | 0.073 | 0 | 1 |  |
| Zscore(K) | 0.256 | -0.42 | 0.675 | 12.284 |
| Zscore(A) | 0.283 | -0.221 | 0.825 | 15.027 |
| Zscore(MA) | 0.156 | -0.77 | 0.443 | 4.563 |
| Zscore(CI) | 0.231 | 2.102 | 0.037 | 9.991 |
